# Characteristics of an In Vitro Mesenteric Lymph Node Cell Suspension Model and Its Possible Association with In Vivo Functional Evaluation

**DOI:** 10.3390/ijms23021003

**Published:** 2022-01-17

**Authors:** Saisai Feng, Jing Li, Dingwu Qu, Fengwei Tian, Leilei Yu, Hao Zhang, Wei Chen, Jianxin Zhao, Qixiao Zhai

**Affiliations:** 1 State Key Laboratory of Food Science and Technology, School of Food Science and Technology, Jiangnan University, Wuxi 214122, China; fengsaisai@outlook.com (S.F.); sun_real@sina.com (J.L.); qudingwu@126.com (D.Q.); fwtian@jiangnan.edu.cn (F.T.); edyulei@126.com (L.Y.); zhanghao61@jiangnan.edu.cn (H.Z.); chenwei66@jiangnan.edu.cn (W.C.); zhaojianxin@jiangnan.edu.cn (J.Z.); 2 School of Food Science and Technology, Jiangnan University, Wuxi 214122, China; 3 National Engineering Research Center for Functional Food, Jiangnan University, Wuxi 214122, China; 4 Wuxi Translational Medicine Research Center and Jiangsu Translational Medicine Research Institute Wuxi Branch, Wuxi 214000, China

**Keywords:** in vitro MLN model, TNBS, TLR2, *A. muciniphila*, *C. butyricum*

## Abstract

In a previous study, we uncovered three immune-responsive patterns of gut microbes using an in vitro mesenteric lymph node cell suspension model, abbreviated as the MLN model hereafter. We used *Akkermansia muciniphila* and *Clostridium butyricum* as the first group directly inducing an immune response, *Bifidobacterium* sp. and *Bacteroides* sp. as the second group evoking an immune response with the help of stimuli (anti-CD3 and anti-CD28 antibodies), and *Lactobacillus* sp. as the third group blunting the immune response with or without stimuli. Our group previously clarified the immune-activation characteristics of *A. muciniphila* and linked its in vivo immune induction effect in GF and SPF mice under homeostasis. In the present study, we supplemented the characteristics of *C. butyricum* and *B. bifidum* in the in vitro MLN model and addressed the specific elements of the model. Finally, we used an in vivo TNBS-challenge model to show the functional differences between these species with different response patterns in vitro. The results showed that *C. butyricum* and *B. bifidum* evoked an immune response in vitro in a dose-dependent and strain-unique manner. Although TLR2, rather than TLR4, is indispensable for immune activation in the present in vitro model, it may not involve interaction between TLR2 and bacterial ligands. Like the PBMC model, the present in vitro MLN model is highly dependent on cell resources and should be given more attention when used to conduct a quantitative comparison. Finally, a mixture of two strong immunogenic strains, *A. muciniphila* and *C. butyricum*, significantly increased the mortality of TNBS-challenged (2,4,6-trinitrobenzene sulfonic acid, TNBS) mice, indicating a possible link between the in vitro MLN model and in vivo functional evaluation. However, more evidence is needed to clarify the associations and underlying mechanisms.

## 1. Introduction

The gut microbiome plays a key role in both local and systemic immune homeostasis. In recent years, it has been shown that both active metabolites and microbes synergistically play immune-regulation roles through cell-surface components [1,2,3,4,5,6,7]. Metabolically, short fatty acids have been reported to strengthen the gut immune response through fueling the cells, interacting with G-protein-coupled receptors, and regulating activities of histone deacetylase or acetylase [2,8]. Bile acids or tryptophan-derived indole derivatives can also affect gut immunity [1,9,10]. The most convincing evidence was derived from germ-free (GF) mice colonized with altered Schaedler flora [11]. This mixture of representative gut microbial species induced activation and de novo generation of colonic T regulatory cells. Specific species in the gut have also been emphasized for their roles in inducing an adaptive immune response. Segmented filamentous bacteria (SFB) in the small intestine of mice induced an antigen-specific Th17 response and protected the host against pathogenic threats [12]. Both *A. muciniphila* and a mixture of *Clostridium* spp. induced a T-cell-dependent immune tolerance in the gut [6,7].

Considering the significance of the gut microbiome in educating a host immune response, numerous investigations and attempts have been devoted to uncovering the diverse effects of gut species. Traditionally, various in vitro and in vivo models have been used to compare the immune regulatory roles of different bacterial strains. In vitro models include the macrophage RAW 264.7, human peripheral blood mononuclear cells (PBMCs), and bone-marrow-derived dendritic cells (BMDCs) [13,14,15]. Although these models are classically for primary immune evaluation, they have limitations. Human PBMCs are not always available, and RAW 264.7 cells are not suitable for evaluating the adaptive immune response. A model combining BMDCs and naïve CD4 + T cells (isolated from splenic cells) was used to evaluate the differentiation of T helper cells. However, this model requires a long experimental period and is not effective enough to screen a large number of samples. The most widely used in vivo immune disorder model is the adoptive transfer model of colitis. Other in vivo models include trinitrobenzesulfonic acid (TNBS) colitis and dextran sulfate sodium (DSS) colitis [16,17].

Previously, Im et al. adopted in vitro mesenteric lymph nodes (MLNs) to screen probiotics with potential immune-regulatory effects [4,18]. We recently reproduced this model in our laboratory and obtained some interesting findings (unpublished work). We uncovered three genus- and species-dependent responsive patterns in the MLN model. We classified *A. muciniphila* and *C. butyricum* as the first group directly inducing an immune response, *Bifidobacterium* sp. and *Bacteroides* sp. as the second group evoking an immune response with the help of stimuli (anti-CD3 and anti-CD28 antibodies), and *Lactobacillus* sp. as the third group blunting the immune response with or without stimuli. In addition, T cell-dependent B cell activation and antibody production are inherent to this model, and the level of IL-10 in the co-culture supernatant can be used to indicate the strength of immune activation. In vivo specific pathogen-free (SPF) and GF mouse experiments under homeostasis showed a consensus result for these strains. Therefore, we believe that this in vitro MLN model may be useful for evaluating the immune-stimulation effect of gut microbial species.

In the present study, we aimed to further investigate the characteristics of the present model as well as the underlying mechanism. In addition, we used an in vivo TNBS-induced mouse model to infer a correlation between their physiological effects in vivo and their roles in vitro. In this way, we hope that this in vitro MLN model can be used to screen for strain combinations with different immuno-regulatory effects.

## 2. Results

### 2.1. Characteristics of Immune Activation of C. butyricum and B. bifidum in an In Vitro MLN Model

**Figure 1:**
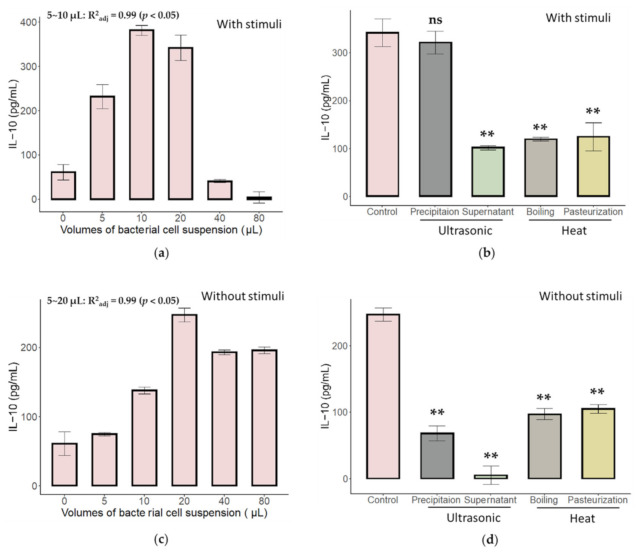
Characteristics of *B. bifidum* and *C. butyricum* in the in vitro MLN model. Dose-dependent response for *B. bifidum* (**a**) and *C. butyricum* (**c**). Adjust R square was calculated within the linear range. The influence of different treatments of bacterial cells of *B. bifidum* (**b**) and *C. butyricum* (**d**) on the immune response. ** Indicated a significant difference (*p* < 0.01) when compared to the control group using a *t*-test. In a commonly used co-culture system (20 μL of bacterial cells co-cultured with 180 μL of MLN cells), the ratio of these cells was 10:1 with 2 × 10^5^ MLN cells. All tests were replicated three times. ns: no significance.

Our previous study showed that the cell surface proteins of *A. muciniphila* AH39 evoked an immune response in the present in vitro MLN model (unpublished work). Although we also found that *C. butyricum* strains showed a similar effect, their dose-dependent effects and heat sensitivity have not been evaluated. The attributes of *B. bifidum* were found to be the same. Thus, we re-evaluated the characteristics of *C. butyricum* and *B. bifidum* with and without stimuli (anti-CD3 and anti-CD28 antibodies), respectively. The results are presented in Figure 1. As indicated, both *B. bifidum* JSNJJNM2 (Bb) and *C. butyricum* FHLJZD47T10 (Cb) showed a dose-dependent immune activation effect. The active substances of these two strains are heat-sensitive because boiling and pasteurization compromised the secretion of IL-10. However, the active substance of Bb is tightly attached to the cell surface because, after ultrasonic treatment, the resuspension of the precipitation component showed an activity level comparable to that of the whole bacterial cell suspension. This is not the same for Cb. Both the resuspension of the precipitant and supernatant had near to no activity after ultrasonic treatment, indicating that the active substance of Cb relied on its spatial structure, which may be provided by anchors to the bacterial cell surface. Finally, it is worth mentioning that most differences in the immune-activation effect of Bb and Cb are still based on whether or not they require stimuli (anti-CD3 and anti-CD28 antibodies). Cb can directly activate the immune response, while Bb can only achieve it with the help of stimuli, as demonstrated in our previous unpublished study.

### 2.2. TLR2, but Not TLR4, Is Indispensable to the Activation of the In Vitro MLN Model

**Figure 2:**
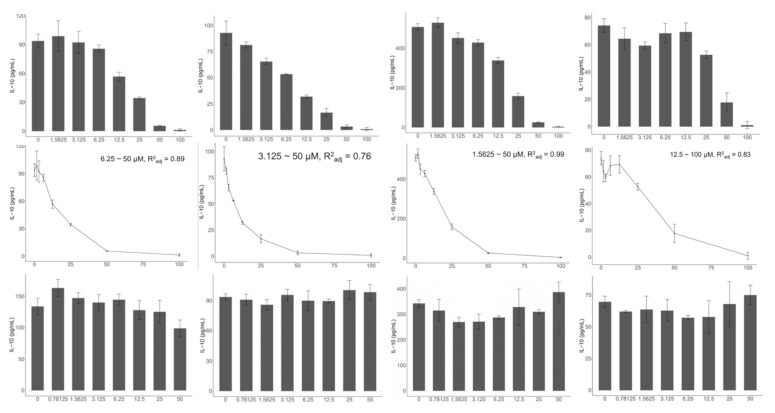
TLR2-dependent immune activation in the in vitro MLN model. Horizontal axis indicates the concentrations of inhibitors (μM) (C29: TLR2 inhibitor; TAK242: TLR4 inhibitor); vertical axis represents the levels of IL-10 in the co-culture supernatant. Adjust R square was calculated within the linear range. All tests were replicated three times.

Our previous study demonstrated that the secretion of IL-10 in the in vitro MLN model is a process involving both bacterial cells that induce innate and adaptive immune activation. T cell receptors recognize cognate protein epitopes on the bacterial cell surface. Other bacterial antigens, such as polysaccharides, teichoic acid, and lipids, activate germline-encoded pattern-recognition receptors (PRRs). Both TLR2 and TLR4 are important PRRs that are mainly involved in the recognition of cell surface polysaccharides and lipids. Therefore, we used TLR2 and TLR4 inhibitors (C29, TAK242) to investigate the necessity of these two receptors in the in vitro MLN model. Interestingly, we found that all strains tested (*A. muciniphila AH39*, *B. bifidum* JSNJJNM2, and *C. butyricum* FHLJZD47T10) under all tested conditions (with or without stimuli) showed similar results (Figure 2). Immune activation in the in vitro MLN model relied on TLR2 rather than TLR4. In addition, it is noteworthy that the control group, in which MLN cells were treated by stimuli and without bacterial cells, showed a similar trend, indicating that the inherent activation strength of TLR2 is necessary in the present model and may be independent of bacterial cells.

### 2.3. Cell Resources Exert an Effect on Bacterial-Induced Responsive Strength

**Figure 3:**
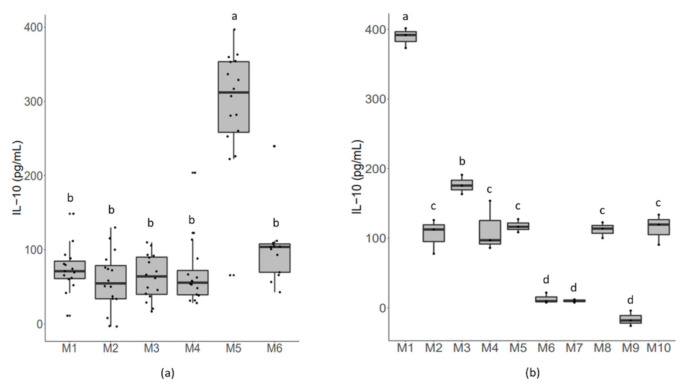
Mouse-dependent responsive strength in the in vitro MLN model. (**a**) Levels of IL-10 in the co-culture supernatant of different *B. bifidum* strains (*n* = 86) and MLN cells isolated from six different C57BL/6J mice. (**b**) Levels of IL-10 in the co-culture supernatant of *A. muciniphila* AH39 and MLN cells isolated from ten different C57BL/6J mice. Different letters indicate significant differences when tested by Tukey’s HSD. All tests were replicated three times.

When comparing the strain-level differences of *B. bifidum* in the present model, we found that cell resources made a significant difference in the level of IL-10 under stimuli-supplemented conditions (Figure 3a). As indicated, although the activation strength of *B. bifidum* showed a strain-level difference, cell resources were still a major factor. Among the tested mice, Mouse 4 had significantly higher IL-10 levels. To verify this effect, we compared the cells isolated from another ten mice and co-cultured them with the same strain, *A. muciniphila* AH39, without stimuli. The results are shown in Figure 3b. Similarly, we found that for the same bacterial strain, the secretion of IL-10 could be classified into three patterns: low (Mouse 9, Mouse 7, Mouse 6); medium (Mouse 2, Mouse 3, Mouse 4, Mouse 5, Mouse 8, Mouse 10); high (Mouse 1). These results indicate the importance of MLN cell resources for the quantitative evaluation of strain-induced immune activation.

### 2.4. Administering a Mixture of A. muciniphila and C. butyricum Significantly Increased the Mortality of TNBS-Challenged Mice

**Figure 4:**
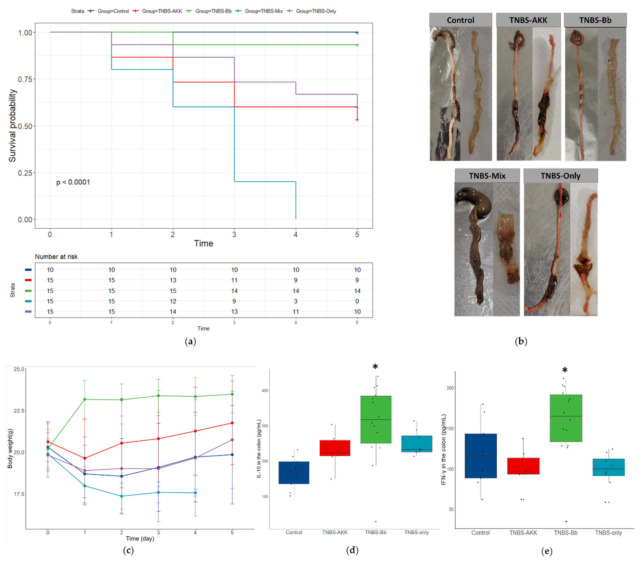
The administration of a mixture of *A. muciniphila* and *C. butyricum* significantly increased the mortality of TNBS-challenged mice. (**a**) Survival curve. (**b**) Changes to gross intestines on the fifth day after the TNBS challenge. (**c**) Changes in mouse body weight. (**d**,**e**) Levels of cytokines (IL-10, IFN-γ) in colon tissue on the fifth day after the TNBS treatment. No statistically significant differences were observed among groups in (**b**–**e**) when tested by Tukey’s HSD. Data about cytokine levels in the TNBS-Mix group were missing for their 100% death rate on the fifth day. * *p* < 0.01.

Due to the specificity of, and differences between, *A. muciniphila*, *C. butyricum*, and *B. bifidum* in the in vitro MLN model, we also compared their effects in an in vivo TNBS-challenged model (Figure 4). As indicated, the administration of Bb produced a significant protective effect, including a significantly higher survival rate, reduced body weight, improved body weight recovery, and almost indiscernible tissue damage in the colon. Mice administered *A. muciniphila* showed a similar or worse degree of disease compared with mice in the TNBS group. Moreover, it is noteworthy that the administration of a mixture of *A. muciniphila* and *C. butyricum* significantly increased the mortality of TNBS-challenged mice. When comparing the cytokine levels in the colon, mice in the Bb group showed a significant increase in IL-10 and IFN-γ when compared to mice in the control or TNBS group.

## 3. Discussion

This study demonstrates a dose-dependent immune activation effect of *B. bifidum* and *C. butyricum*. Together with *A. muciniphila*, these strains showed distinct immune activation effects. For *A. muciniphila*, our previous study showed that cell surface protein as a cognate T cell receptor antigen can directly activate the immune response in an in vitro MLN model. However, there was a different situation for *C. butyricum*. Although *C. butyricum* can also directly activate the immune response, whole bacterial cell proteins of *C. butyricum* failed to evoke a comparable immune response (data not shown). Both the supernatant and precipitant had almost no activity after ultrasonic treatment. We speculate that the anchoring status of active substances may be necessary for the immune activation effect. Erturk-Hasdemir et al. [19] identified that PSA and its covalently anchored bacterial outer membrane lipids are both required for the activation of antigen-presenting cells [19].

The importance of TLR2 and TLR4 in the recognition of micro-organism antigens has been widely reported [4,20,21,22]. Although TLR2 rather than TLR4 is necessary for immune activation in the present MLN models, the interaction between TLR2 and bacterial antigens may not have induced the production of IL-10. In the control group supplemented with stimuli (anti-CD3 and anti-CD28 antibodies) rather than bacterial cells, the production of IL-10 was also inhibited by the inhibitor of TLR2 and with a similar range of inhibitor concentrations. Therefore, we inferred that the inherent background level of TLR2 signaling was indispensable for immune activation in this in vitro MLN model. Generally, TLR4 senses bacterial ligands, including lipopolysaccharides (LPS), cell surface proteins, etc. [23,24]. For example, an outer membrane protein Amuc_1100 has been demonstrated to interact with TLR4 and active a TLR4-dependent NF-κB signaling pathway [24]. However, we propose a non-TLR4-dependent immune activation in the present in vitro model. TLR4 may be expressed at low levels in these immune cells, and other types of innate immune responses may mainly induce these effects, such as recognition of bacterial cell surface polysaccharides by a complementary pathway. In addition, we also found that individual differences in mice had a great influence on the level of IL-10, which was even greater than the differences induced by different strains. These in vitro immunomodulatory evaluation models are not surprising. Basal gene expression in peripheral blood mononuclear cells also shows a high degree of individual variance [21], which may be caused by the varied subpopulation composition of lymphocytes among the different mice [25].

The in vivo TNBS model has often been used to mimic the pathogenesis of Crohn’s disease and to screen protective compounds with therapeutic or preventive effects [26]. Owing to its efficiency and easy conduciveness, it is especially convenient for screening functional probiotics and prebiotics with anti-inflammatory effects [27,28,29]. However, it was not an easy model to implement due to the high mortality and the severe tissue injury that might obscure the experimental results. In our study, we found that except for Bb and the control group, most mice in other groups had no diarrhea or bloody stools in the first two days after the TNBS challenge. These mice often had intestinal obstruction due to toxicity and damage of the TNBS ethanol solution on gut nerve cells, which has also been documented by Ozaki et al. in 2003 [30]. Second, significantly increased IL-10 and IFN-γ levels were only found in the Bb group. Neither the TNBS nor the AKK group had a higher level of cytokines when compared to the control group. We inferred that serious weight loss in these groups after the TNBS challenge resulted in a fasting status, which may have blunted the immune response. A previous study showed that a 24 h fast in humans is sufficient to blunt CD4+ T helper cell responsiveness, offering credibility for our hypothesis [31]. Third, we found that administration of a mixture of *A. muciniphila* and *C. butyricum* significantly increased mortality in mice. Our previous study showed that both *A. muciniphila* and *C. butyricum* can directly activate the immune response in an in vitro model; in particular, *A. muciniphila* had a weak immune-activation effect in both GF and SPF mice under homeostasis (unpublished data). Both *A. muciniphila* and *C. butyricum* induce colonic T regulatory cells [6,7,32]. Mechanistically, cell surface proteins of *A. muciniphila* are more immunogenic and may induce adaptive immune responses in a context-dependent manner even under homeostasis status [32]. Moreover, the specific role of *A. muciniphila* in gut immunity may also explain its enhancing effect in anti-PD-1 based immunotherapy against tumors [33]. However, it is worth mentioning that neither a single strain of *A. muciniphila* nor *C. butyricum* is capable of inducing a strong immune response in conventional mice [7,32]. Therefore, a synergistic effect of these specific gut microbes may play a significant role in shaping the unique gut immune environment. We believe that a mixture of *A. muciniphila* and *C. butyricum* might evoke an enhanced gut immune response during the initial hours after the TNBS challenge and then aggravate the mortality in this group. Meroni et al. observed severe infiltration of neutrophils and monocytes 6 h after rectal administration of oxazolone [34]. However, we did not test the immunity strength in the different groups during the first several hours. Thus, the present in vivo study provided the first evidence of the possible immune activation roles of *A. muciniphila* and *C. butyricum* under an acute TNBS-challenged status and was accidentally in accordance with the results of in vitro MLN models, which also indicate a specific immune regulatory role of these two species. However, more evidence is needed to demonstrate and clarify the possible links between in vitro and in vivo models.

Recently, a research hotspot of the gut microbiome is as an adjuvant of immunotherapies, targeting anti-tumor immunity, colitis, and other associated diseases [35]. Investigations starting with immuno-phenotype or bacterial-derived active molecules would be helpful to uncover a clear and precise interaction landscape and to finally guide microbial design. The present in vitro MLN cell suspension model with a high degree of effectiveness in identifying a genus- or species-dependent immune response pattern may be promising for these attempts.

## 4. Materials and Methods

### 4.1. Strains, Media, and Growth Conditions

*A. muciniphila* AH39 was cultured in BHI broth supplemented with 0.25% porcine gastric mucin (type III, 81 Sigma-Aldrich) for 72 h. *C. butyricum* FHLJZD47T10 was cultured in BHI broth for 24 h. *B.* *bifidum* JSNJJNM2 and another 85 *B. bifidum* strains were cultured in MRS broth for 24–48 h. All bacteria were grown in an anaerobic chamber (Electrotek, Shipley, UK) with the following gaseous characteristics: 5% hydrogen, 5% 85 carbon dioxide, and 90% nitrogen. For in vitro MLN model, freshly cultured bacterial cells were collected and washed with phosphate-buffered saline (PBS). After that, bacterial cells were re-suspended and adjusted to 0.5 of optical density at 600 nm for further experiments. For in vivo MLN experiment, bacterial cells were collected and concentrated to 5 × 10^9^ CFU/mL for oral administration.

### 4.2. In Vitro MLN Co-Culture Model

An in vitro MLN co-culture model was developed according to the reports of Im et al. [18]. MLN cells were prepared as follows: freshly isolated MLNs from 6 weeks of male C57BL/6 mice were mechanically disrupted. MLN cells were cultured in RPMI 1640 medium (Thermo Fisher Scientific, Grand Island, NY, USA) supplemented with 10% FBS (Hyclone, Smithfield, Australia), 10 mM HEPES (Sigma-Aldrich, St. Louis, MO, USA), 100 U/mL penicillin (Sigma-Aldrich, St. Louis, MO, USA), 100 U/mL streptomycin (Sigma-Aldrich, St. Louis, MO, USA), 0.05 mM 2-beta-mercaptoethanol (Sigma-Aldrich), 3 mM glutamine, and 150 μg/mL gentamicin (Sangon Biotech, Shanghai, China). Except for the dose-dependent experiments, the MLN cell suspension (180 μL) containing 2 × 10^5^ cells was plated in a 96-well plate and co-cultured with 20 μL of the bacterial suspension (OD600 0.5; bacterial cell concentration: ~1 × 10^8^ CFU/mL; with a ratio of 1:10 for cells and bacterial colony forming units) for 72 h with or without 1 mM anti-CD3 (eBioscience, San Diego, CA, USA) and 1 mM anti-CD28 (eBiosience, San Diego, CA, USA). IL-10 in the cell supernatant was quantified using a mouse ELISA kit (eBioscience, San Diego, CA, USA) according to the manufacturer’s protocols.

For the dose-dependent experiments, bacterial cells were added at doses of 0, 5, 10, 20, 40, and 80 μL. For heat sensitivity and localization experiments, freshly obtained bacterial cell suspensions (OD600 0.5) were divided into equal 1 mL aliquots. First, one milliliter of bacterial cells was treated by probe sonication at 81 W for 4 min (3 s with a 3 s interval on ice). Next, the bacterial cell suspensions were centrifuged for 5 min at 10,000× *g*. The supernatant was obtained, and the precipitate was re-suspended in 1 mL of PBS. Then, one milliliter of the previously prepared fresh bacterial cell suspension was boiled in a thermostatic metal bath at 100 °C for 15 min and immediately cooled on ice. Finally, a relatively mild treatment was performed. One milliliter of bacterial cell suspension was pasteurized at 65 °C for 30 min and cooled immediately on ice.

To investigate the effect of TLR2 and TLR4 in the present in vitro MLN model, inhibitors of TLR2 (C29) and TLR4 (TAK242) were purchased from MedChemExpress [36,37]. Inhibitors were diluted and added to the co-culture system. For C29, we tested concentrations ranging from 0.0625 to 4 μM. For TAK242, we tested concentrations ranging from 0.03125 μM to 2 μM.

All tests in the in vitro MLN model were replicated three times.

### 4.3. Animal Experiment

Six-week-old male BALB/C mice were obtained from Vital River Limited Company (Beijing, China). The mice were fed under SPF conditions at the animal facility of Jiangnan University. A total of 70 mice were randomly divided into 5 groups, with 15 mice in the TNBS, AKK, Bb, and Mix groups and 10 mice in the control group. After a week of flexible feeding, mice were orally administered 200 μL of PBS or freshly prepared bacterial suspension (containing 1 × 10^9^ bacterial cells) for another four days. On the fifth day, the mice were exposed to TNBS rectally at a concentration of 25 μg/μL in 35% ethanol (50 μL per mouse). After the TNBS challenge, mice were continuously administered bacterial suspension (containing 1 × 10^9^ bacterial cells) or PBS for another five days. The general condition of the mice was recorded. Finally, on the fifth day after the TNBS challenge, mice were euthanized via inhalation of 100% CO_2_, and the colon was removed for quantification of cytokines (IL-10 and IFN-γ) using a mouse ELISA kit (eBioscience, San Diego, CA, USA) according to the manufacturer’s protocols.

### 4.4. Statistical Analysis and Data Visualization

Statistical analysis and data visualization were performed using R v4.1.1. Survival analysis was performed using the R package, Survminer [38]. Survival curves were visualized using ‘ggplot2’ version 0.4.9. (https://CRAN.R-project.org/package=survminer, accessed on 17 October 2021). Multiple comparisons were performed using Tukey’s HSD. For concentration-associated series tests, linear regression analysis was performed. Pictures were obtained from Servier Medical Art (https://smart.servier.com/, accessed on 17 October 2021).

## 5. Conclusions

This study investigated the characteristics of the in vitro MLN model. We found that immune activation with *C. butyricum* and *B. bifidum* was dose-dependent in the in vitro model and had unique responsive characteristics. Although TLR2 (rather than TLR4) is indispensable for immune activation in the present in vitro model, it may involve an interaction process between TLR2 and bacterial ligands. Similar to the PBMC model, the present in vitro MLN model has high individual variance and should be given more attention when used to quantitatively compare the immune-regulatory effect among different strains. Finally, a mixture of *A. muciniphila* and *C. butyricum* significantly increased mortality in TNBS-challenged mice. We inferred that a mixture of *A. muciniphila* and *C. butyricum* together evoked an enhanced gut immune response during the initial hours after the TNBS challenge and then aggravated the mortality in this group. This observation is in accordance with the strong immunogenic roles of these two strains in the in vitro MLN model, indicating a possible association between the in vitro MLN model and the in vivo functional evaluation. However, more evidence is needed to definitively link this in vitro model and various in vivo inflammatory models and to clarify the underlying mechanisms.

## Data Availability

The data presented in this study are available on request from the corresponding author.
